# The effects of childhood maltreatment on epigenetic regulation of stress-response associated genes: an intergenerational approach

**DOI:** 10.1038/s41598-018-36689-2

**Published:** 2019-04-18

**Authors:** Laura Ramo-Fernández, Christina Boeck, Alexandra M. Koenig, Katharina Schury, Elisabeth B. Binder, Harald Gündel, Jöerg M. Fegert, Alexander Karabatsiakis, Iris-Tatjana Kolassa

**Affiliations:** 1 Department of Clinical & Biological Psychology, Institute of Psychology and Education, Ulm University, Albert-Einstein-Allee 47, Ulm, 89081 Germany; 2 Department of Translational Research in Psychiatry, Max Planck Institute of Psychiatry, Munich, 80804 Germany; 3 Department of Psychiatry and Behavioral Sciences, Emory University School of Medicine, Atlanta, GA 30322 USA; 4 Department of Child and Adolescent Psychiatry and Psychotherapy, University Hospital Ulm, 89075 Ulm, Germany; 5 Department of Psychosomatic Medicine and Psychotherapy, University Hospital Ulm, 89081 Ulm, Germany

**Keywords:** Epigenetics, Gene regulation, Translational immunology, Psychology

## Abstract

While biological alterations associated with childhood maltreatment (CM) have been found in affected individuals, it remains unknown to what degree these alterations are biologically transmitted to the next generation. We investigated intergenerational effects of maternal CM on DNA methylation and gene expression in *N* = 113 mother-infant dyads shortly after parturition, additionally accounting for the role of the *FKBP5* rs1360780 genotype. Using mass array spectrometry, we assessed the DNA methylation of selected stress-response-associated genes (FK506 binding protein 51 [*FKBP5*], glucocorticoid receptor [*NR3C1*], corticotropin-releasing hormone receptor 1 [*CRHR1*]) in isolated immune cells from maternal blood and neonatal umbilical cord blood. In mothers, CM was associated with decreased levels of DNA methylation of *FKBP5* and *CRHR1* and increased *NR3C1* methylation, but not with changes in gene expression profiles. Rs1360780 moderated the *FKBP5* epigenetic CM-associated regulation profiles in a gene × environment interaction. In newborns, we found no evidence for any intergenerational transmission of CM-related methylation profiles for any of the investigated epigenetic sites. These findings support the hypothesis of a long-lasting impact of CM on the biological epigenetic regulation of stress-response mediators and suggest for the first time that these specific epigenetic patterns might not be directly transmitted to the next generation.

## Introduction

**Figure 1:**
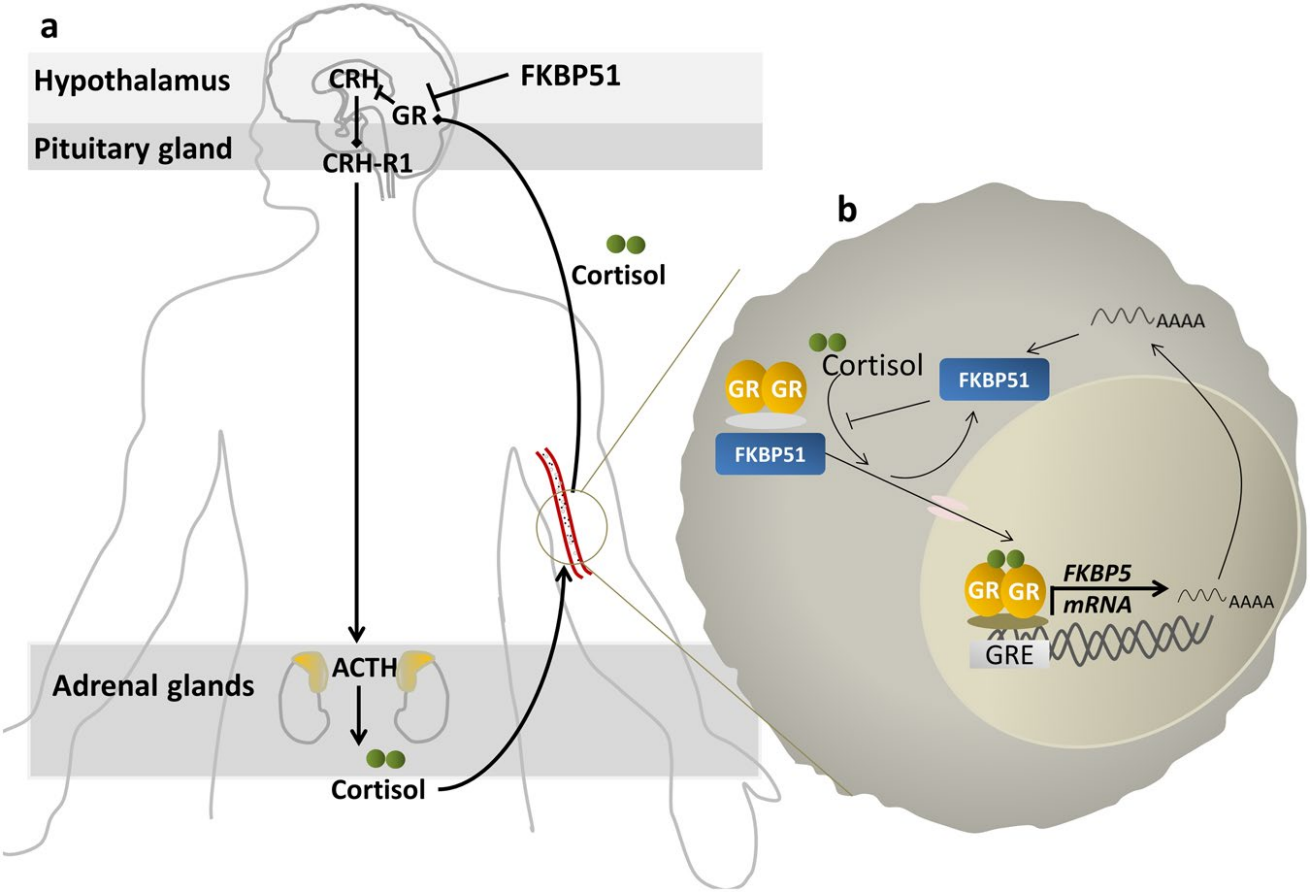
Schematic representation of the HPA axis and its link to the immune system. (**a**) Upon acute stress exposure, the hypothalamus releases the neurohormone corticotropin release hormone (CRH) into the blood stream and eventually binds, among others, to the receptor 1 for corticotropin release hormone (CRH-R1) in the anterior pituitary glands. The complex CRH/CRH-R1 initiates the peripheral stress response by inducing the release of adrenocorticotropic hormone (ACTH). ACTH stimulates the secretion of cortisol from the adrenal cortex into the peripheral blood stream. Binding of peripheral cortisol to central glucocorticoid receptor (GR), expressed within the hypothalamus and the pituitary gland, induces a negative feedback loop that prevents the continuous secretion of glucocorticoids^60^. The cortisol-GR complex is additionally influenced by its co-chaperone FKBP51, which reduces GR sensitivity and thereby diminishes the GR-induced negative feedback inhibition of peripheral cortisol release^61^. (**b**) Tandem GR and FKBP51 regulation: On the one hand, the *FKBP5* regulatory genetic region includes several glucocorticoid response elements (GREs) where GR, when bound to a glucocorticoid molecule, can directly activate *FKBP5* transcription^61^. On the other hand, binding of FKBP51 to the GR reduces GR affinity to cortisol and inhibits its translocation into the nucleus^61^. Both mechanisms contribute to an ultra-short feedback loop, promoting GR resistance.

Childhood maltreatment (CM) is so far an underestimated global phenomenon present in all societies and social classes. CM comprises experiences of physical, sexual and emotional abuse, as well as physical and emotional neglect during childhood and adolescence and constitutes a major threat to the child’s mental and physical development with long-term consequences for both mental and somatic health^1–4^. The epigenetic alterations in DNA methylation occurring in the aftermath of CM are pivotal for the adaptation to the early life environment^5^, and can thereby affect gene expression levels^6^ and molecular responses to environmental stressors. Epigenetic alterations within key player genes of the hypothalamic-pituitary-adrenal (HPA) axis, the main coordinator of the physiological stress response (Fig. 1), are discussed to biologically contribute to health consequences observed in CM-affected individuals^7,8^.

Accordingly, mounting evidence suggests that CM is associated with alterations in DNA methylation within the glucocorticoid receptor gene (*NR3C1)*^9–13^ and its regulatory co-chaperone *FK506-binding protein 51* (FKBP51), which is encoded by the *FKBP5* gene^13,14^. As depicted in Fig. 1, a balanced regulation between the GR and FKBP51 is essential for a normal physiological stress response. Another important regulator of HPA axis activity is the corticotropin-releasing hormone receptor 1 (CRH-R1, codified by the *CRHR1* gene; Fig. 1)^15,16^. While rodent studies demonstrated an association between chronic mild stress and *CRHR1* hypermethylation^17^, changes in epigenetic regulation within *CRHR1* associated with CM in humans have not been investigated so far.

Besides the environmental exposure to CM, individual genetic variability might further account for vulnerability to health outcomes. Here, a single nucleotide polymorphism within the gene *FKBP5* (rs1360780; T/C alleles) has gained scientific attention. Investigating gene × environment interactions, the rs1360780 T allele has been shown to interact with early life trauma to decrease *FKBP5* methylation^14^, increase GR resistance^14^, and to predict adult psychopathology^14,18,19^.

Not only individuals who experienced CM, but also their offspring show an increased lifetime risk for stress-related behavioral^20–22^ as well as physiological disorders (e.g. asthma or allergies)^23^. Moog *et al*.^24^ further provided first evidence that newborns already show biological consequences of maternal CM and present with smaller brain size and lower grey matter volume^24^. While rodent studies suggested that epigenetic alterations associated with early life adversity might be stably inherited through the germ cells^25–27^, evidence for an intergenerational transmission of CM consequences^28^ via germ cells in humans is lacking so far.

We hypothesized that CM-associated epigenetic alterations involved in stress reactivity are directly transmitted to the next generation. To test this, we assessed DNA methylation and gene expression profiles of selected stress-response related genes, namely *FKBP5*, *CRHR1*, and *NR3C1* in peripheral and umbilical cord blood cells from mothers and their newborns, respectively. We additionally accounted for the moderating role of *FKBP5* rs1360780 on the impact of CM on epigenetic changes and genetic regulation.

## Results

### Descriptives

Mothers with CM experiences (CM^+^) and their infants did not differ in age, ethnicity, newborn’s sex, relative blood cell composition, and rs1360780 allelic distribution from mothers without CM experiences (CM^-^) and their infants. For a summary of demographic data see Table 1.

**Table 1:** Demographic characteristics and CM exposure.

	CM^−^	CM^+^	Statistics	*p* ^a^
	(*N* = 59)	(*N* = 58)		
Socio-demographic characteristics				
Mean age (Mean ± SD, years)	33.1 ± 4.2	32.8 ± 4.6	*t*_*(115)*_ = 0.36	0.72
Maternal ethnicity Caucasian^b^ (*N* (%))	59 (100)	56 (96.6)	*χ*^2^_(1)_ = 2.07	0.24
Academic education (*N* (%))	35 (59.3)	28 (48.3)	*χ*^2^_(1)_ = 1.03	0.31
Female sex of infant^c^ (*N* (%))	24 (40.7)	28 (48.3)	*χ*^2^_(1)_ = 0.68	0.41
Gestational and neonatal conditions^c^				
Birth weight (Mean ± *SD*, in *g*)	3460 ± 474	3312 ± 512	*t*_*(111)*_ = 1.48	0.14
Gestational age (in weeks ± SD)	39.7 ± 1.1	39.3 ± 1.6	*t*_*(111)*_ = 1.58	0.12
Caesarean section^d^ (*N* (%))	20 (35.1)	13 (23.6)	*χ*^2^_(1)_ = 3.44	0.27
Smoked during pregnancy (*N* (%))	5 (8.5)	5 (10.3)	*χ*^2^_(1)_ = 0.12	0.73
Blood cell composition				
Maternal relative lymphocyte count (Mean ± SD, %)	18.7 ± 4.7	18.5 ± 5.0	*t*_*(105)*_ = −0.67	0.50
Children relative lymphocyte count (Mean ± SD, %)	30.4 ± 8.2	29.4 ± 7.0	*t*_(89)_ = 0.59	0.55
Maternal relative monocyte count (Mean ± SD, %)	5.8 ± 1.4	6.0 ± 4.7	*t*_*(105)*_ = 0.28	0.78
Children relative monocyte count (Mean ± SD, %)	8.3 ± 2.2	9.3 ± 3.2	*W* = 799.5	0.07
*FKBP5* rs1360780 allelic distribution				
Mother T allele carrier (*N* (%))	20 (33.9)	26 (44.8)	*χ*^2^_(1)_ = 1.04	0.31
Children T allele carrier (*N* (%))	24 (41.4)	26 (47.2)	*χ*^2^_(1)_ = 0.19	0.66
Childhood Maltreatment				
CTQ sum score (Mean ± SD)	27.1 ± 1.9	40.2 ± 12.1	*t*_*(115*)_ = −8.16	<0.001
Emotional abuse^e^ (*N* (%))	—	22 (37.9)		
Physical abuse^e^ (*N* (%))	—	16 (27.6)		
Sexual abuse^e^ (*N* (%))	—	16 (27.6)		
Emotional neglect^e^ (*N* (%))	—	40 (69.0)		
Physical neglect^e^ (*N* (%))	—	10 (17.2)		

Group differences calculated with chi-square tests for binomial and *t-*tests for continuous variables.

SD = Standard deviation; CM = Childhood maltreatment; CTQ = C*hildhood Trauma Questionnaire*; CTQ sum score =  Childhood maltreatment load.

^a^Main effect of the CTQ classification (*t*-tests or chi-square tests).

^b^One study participant of Brazilian origin and one of North-American origin.

^c^For gestational and neonatal characteristics, only mother-infant dyads were included: *N*_*CM-*_ = 58; *N*_*CM*__+_ = 55.

^d^Included planned (*N*_*CM-*_ = 16, *N*_*CM*__+_ = 12) and emergency (*N*_*CM-*_ = 4, *N*_*CM*__+_ = 1) forms of caesarean section.

^e^Amount of women with at least mild experiences in this CTQ subscale.

### Maternal methylation profiling of *FKBP5*, *CRHR1*, and *NR3C1*

**Figure 2:**
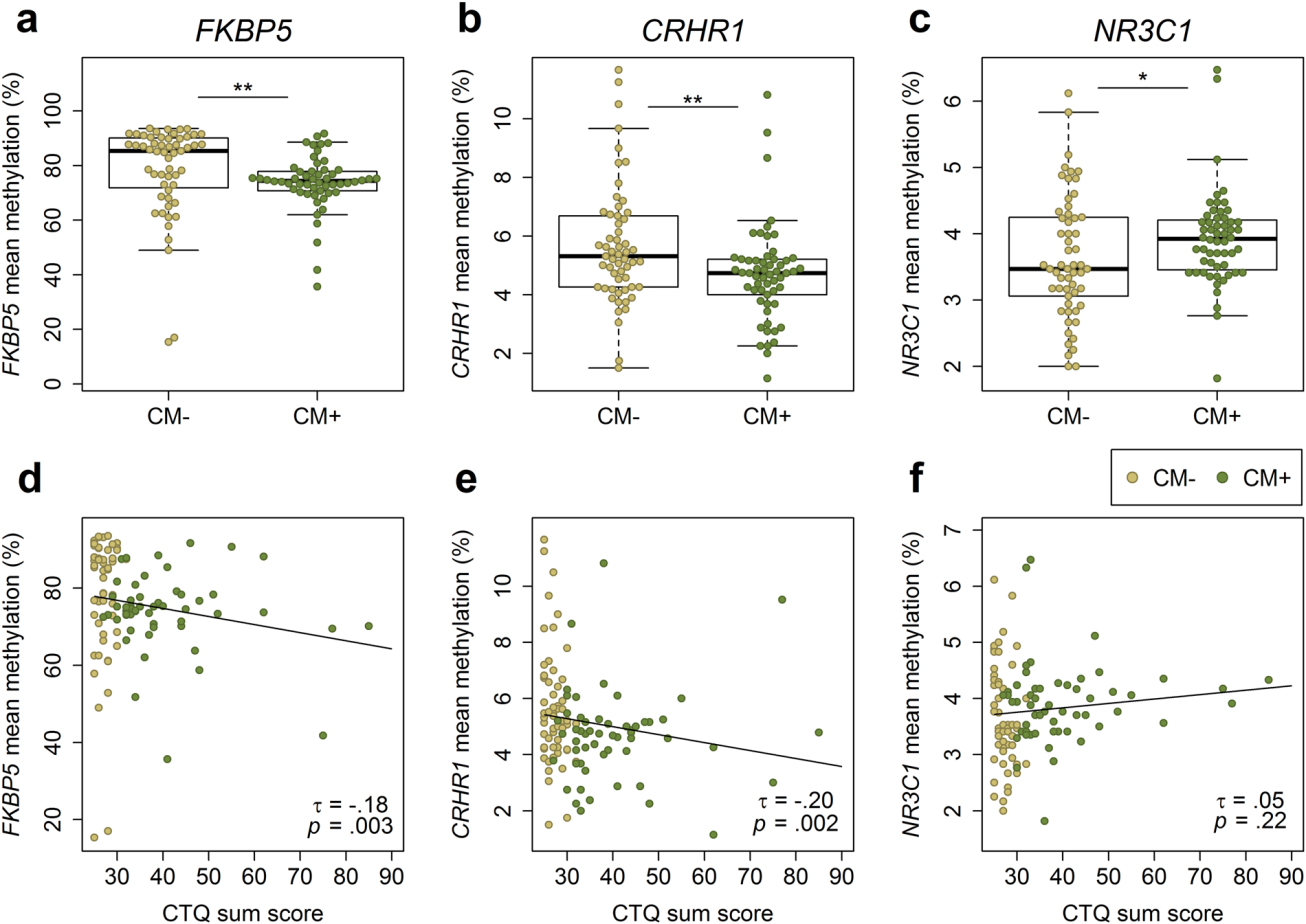
*FKBP5*, *CRHR1*, and *NR3C1* methylation in mothers. (**a**–**c**) Group differences in the DNA mean methylation of the targeted genetic regions. *FKBP5* (*N* = 109) and *CRHR1* (*N* = 114) were hypomethylated in the aftermath of CM experiences while *NR3C1* was significantly higher methylated in women with a history of CM (*N* = 113). (**d–f**) Dose-response effect of CM severity on methylation levels: the higher the CTQ sum score, the lower were the *FKBP5* and *CRHR1* mean methylation levels.

CM^+^ mothers showed lower mean methylation levels within intron 7 of *FKBP5* (73.7% ± 10.2% vs. 78.4% ± 16.8%; *W* = 1994.5, *p* = 0.001, *N* = 109; Fig. 2a) compared to CM^**−**^ mothers. Moreover, there was a significant negative association between mean *FKBP5* methylation and the CTQ sum score, a cumulative measure of the severity of CM experiences (maltreatment load) (*τ* = −0.18, *p* = 0.003, *N* = 109; Fig. 2d). CM^**+**^ mothers also showed less *CRHR1* methylation compared to CM^**−**^ mothers (4.7 ± 1.7% vs. 5.7 ± 2.1%; *W* = 2134, *p* = 0.004, *N* = 114; Fig. 2b) and with increasing maltreatment load, the mean methylation of *CRHR1* decreased (*τ* = −0.20; *p* = 0.002, *N* = 114; Fig. 2e). In contrast, CM^+^ mothers had a higher mean methylation in *NR3C1* exon 1 F compared to CM^**−**^ mothers (3.9 ± 0.7% vs 3.6 ± 0.9%; *W* = 1256, *p* = 0.026, *N* = 113; Fig. 2c), but maltreatment load was not positively correlated with *NR3C1* mean methylation (*τ* = 0.05; *p* = 0.22, *N* = 113; Fig. 2f). When we included maternal age, glucocorticoid medication and the time interval (days) between delivery and PBMC isolation as covariates, the group-wise differences for *NR3C1* (*β* = 0.16, *p* = 0.05, *N* = 113) and *CRHR1* (*β* = *−*0.24, *p* = 0.007, *N* = 114) remained significant, whereas the CM-associated hypomethylation of *FKBP5* (*β* = *−*0.15, *p* = 0.06, *N* = 109) and the negative association between *FKBP5* (*β* = *−*0.14, *p* = 0.08, *N* = 109) and *CRHR1* (*β* = *−*0.15, *p* = 0.09, *N* = 114) methylation and maltreatment load were reduced to a trend. Single CpG unit (fragments with one or more CpG sites, depending on the enzymatic cleavage) analyses are described in SI (section 7 and Table S2).

### Maternal gene expression analyses in peripheral immune cells

As changes in methylation might translate into alterations in gene expression, we next analyzed gene expression levels of *FKBP5* and *NR3C1* in PBMC. No significant difference was found for gene expression of *FKBP5* or *NR3C1* between women with and without CM (Table S3) and we did not find any significant correlations between DNA methylation and gene expression levels, neither for *FKBP5 (τ* = *−*0.11, *p* = 0.21; *N* = 66), nor for *NR3C1* (*τ* = *−*0.08, *p* = 0.34; *N* = 66).

### Comparison of methylation patterns and gene expression in mother-infant dyads

**Figure 3:**
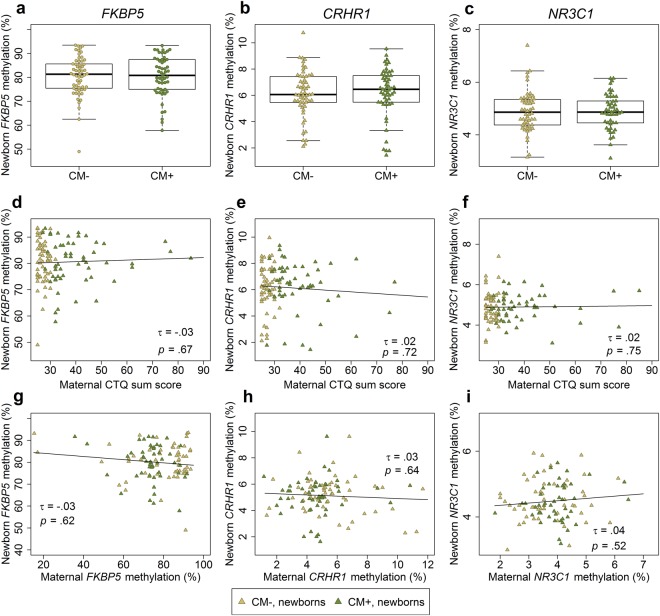
*FKBP5*, *CRHR1*, and *NR3C1* DNA methylation in infants. (**a**–**c**) DNA methylation did not differ statistically between infants from CM^+^ mothers and infants from mothers without CM experiences (*FKBP5: N* = 112, *W* = 1547, *p* = 0.96; *CRHR1: N* = 109, *W* = 1367, *p* = 0.48; *NR3C1*: *N* = 101, *W* = 1264, *p* = 0.95; all analyses conducted two-sided). (**d**–**f**) Severity of CM experiences did not statistically affect infants’ DNA methylation for any of the targeted genes. (**g**–**i**) Newborn’s DNA methylation did not correlate with maternal DNA methylation in any of the three targeted genes. For correlational analyses between mother and infant’s mean DNA methylation, only CpG sites that consistently survived quality criteria in both groups, mothers and infants, were included for analyses.

To test for an intergenerational transmission of the observed DNA methylation patterns, we correlated DNA methylation levels of the targeted genes in mother-infant dyads. Maternal and infant’s methylation levels were not significantly correlated for any of the candidate genes (*FKBP5: τ* = *−*0.03, *p* = 0.69, *N* = 104 dyads; *CRHR1*: *τ* = 0.05, *p* = 0.43, *N* = 112 dyads; *NR3C1: τ* = 0.04, *p* = 0.57, *N* = 93 dyads). Similar to the results in mothers, infants showed no correlation between the levels of DNA methylation and gene expression for *NR3C1* (*τ* = 0.21, *p* = 0.11; *N* = 29) and *FKBP5* (*τ* = −0.14, *p* = 0.26; *N* = 35; Fig. 3). Most importantly, infants of CM^+^ mothers showed no changes in DNA methylation of *FKBP5*, *CRHR1* and *NR3C1* (Fig. 3) or gene expression levels of *FKBP5* and *NR3C1* (Table S3) compared to infants of CM^**−**^ mothers. These findings remained unchanged when we accounted for gestational age at birth and gender of the infants as covariates. Additionally, the methylation analyses of single CpG units showed no significant mother-child correlation for any of the targeted sites (all *p-*values > 0.05).

### The functional role of rs1360780 allelic variation in *FKBP5* and *NR3C1* regulation

**Figure 4:**
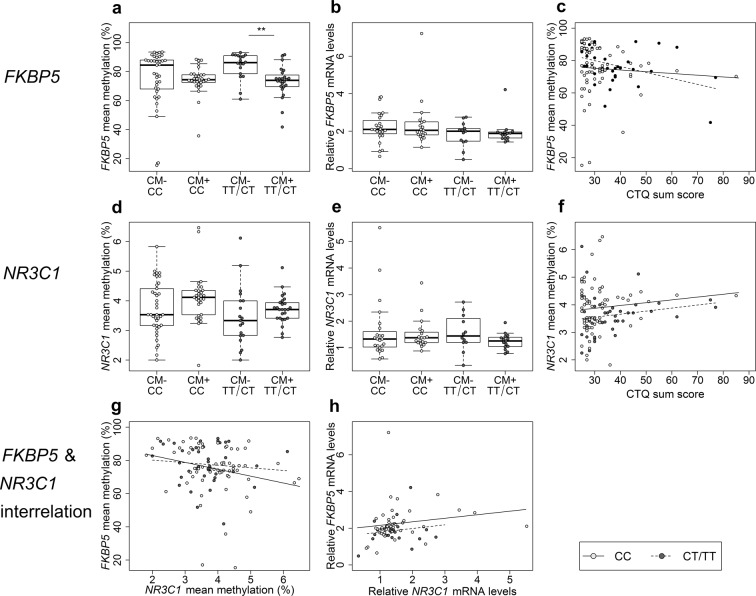
Rs1360780 genotype specific effects on *FKBP5* and *NR3C1* regulation in mothers. (**a**) Rs1360780 genotype dependent alterations in *FKBP5* methylation. Among the T allele carriers, CM^+^ women (*N* = 24) had significantly lower *FKBP5* methylation levels compared to CM^−^ women (*N* = 18) (*W* = 346.5, *p* < 0.001, total *N* = 42). In mothers with the CC genotype, experiencing CM had no effect on *FKPB5* methylation (*W* = 669, *p* = 0.15, *N* = 67). (**b**) *FKBP5* relative gene expression and CM status were not associated, independently of the rs1360780 genotype (CC: *W* = 225, *p* = 0.88, *N* = 42; CT/TT: *W* = 109, *p* = 0.62, *N* = 28). (**c**) Only among the T allele carriers, the hypomethylation of *FKBP5* and the severity of the maltreatment were correlated (*τ* = −0.31, *p* = 0.005, *N* = 41). (**d**) The analysis of the rs1360780 × CM interaction showed that the *FKBP5* genotype does not moderate *NR3C1* methylation (*β* = −0.06, *p* = 0.72, *N* = 113*)* nor (**e**) gene expression (*β* = *−*0.11, *p* = 0.66, *N* = 69). (**f**) The severity of maltreatment experiences was not associated with *NR3C1* methylation, independently of the genotype (CC: *τ* = 0.05, *p* = 0.58, *N* = 68; CT/TT: *τ* = 0.17, *p* = 0.11, *N* = 45). (**g**) The association between the methylation of *NR3C1* and *FKBP5* was genotype-dependent: in CC women, *FKBP5* and *NR3C1* mean methylation were negatively associated (*τ* = −0.18, *p* = 0.03, *N* = 64) but this effect was not observed in T carriers (*τ* = −0.04, *p* = 0.68, *N* = 42). (**h**) Only CC women showed a positive association between relative levels of gene expression of *NR3C1* and *FKBP5* (*τ* = 0.29, *p* = 0.006, *N* = 42; T carriers: *τ* = −0.02, *p* = 0.9, *N* = 27). CM^−^ CC: rs1360780 CC women without a history of CM; CM^−^ CT/TT: T-carriers without a history of CM. CM^+^ CC: CC women with CM experiences; CM^+^ CT/TT: T-carriers with CM experiences. All analyses were tested two-sided.

To investigate whether rs1360780 genotype (C/T) and CM mediated the DNA methylation levels of the target genes, we conducted interaction analyses, which revealed an interaction between *FKBP5* T carrier status and CM on mean *FKBP5* methylation among mothers (*β* = −0.27, *p* = 0.04, *N* = 109). Post hoc analyses showed that, within mothers carrying at least one T allele, CM^+^ mothers had less *FKBP5* methylation than CM^**−**^ mothers (*W* = 346.5, *p* < 0.001, *N* = 42; Fig. 4a). CM^+^ and CM^**−**^ mothers homozygous for the C-allele did, however, not differ significantly with respect to *FKBP5* methylation levels (*W* = 669, *p* = 0.15; *N* = 67; Fig. 4a). *FKBP5* gene expression was not altered depending on the rs1360780 genotype (Fig. 4b). Moreover, only in T allele carriers was a negative association between *FKBP5* methylation levels and the CTQ sum score found (*τ* = −0.31, *p* = 0.005, *N* = 42; Fig. 4c). In contrast, there was no CM × rs1360780 interaction effect on maternal *NR3C1* methylation (*β* = −0.06, *p* = 0.72, *N* = 113; Fig. 4d–f). In infants, maternal CM experiences did not interact with the infant’s rs1360780 genotype on either *FKBP5* (*β* = −0.13, *p* = 0.41, *N* = 113) or *NR3C1* mean methylation levels (*β* = 0.06, *p* = 0.71, *N* = 103).

Based on the T allele and CM-dependent effects on *FKBP5* mean methylation, we next investigated whether the regulatory interrelation between *FKBP5* and *NR3C1* might also be influenced by the *FKBP5* rs1360780 genotype. Indeed, we observed an allele-specific effect of rs1360780 on the association between *NR3C1* and *FKBP5*: only women homozygous for the C allele showed a negative correlation between *NR3C1* and *FKBP5* mean methylation (*τ* = −0.18, *p* = 0.03, *N* = 64; Fig. 4g), and a positive correlation between *NR3C1* and *FKBP5* gene expression levels (*τ* = 0.29, *p* = 0.006, *N* = 37; Fig. 4h), while none of these associations were present among T carriers (Fig. 4g–h).

## Discussion

The aim of this study was to investigate the intergenerational impact of CM on gene regulation of stress-response associated genes in mother-infant dyads. Our results suggest that CM induces long-lasting alterations in the DNA methylation pattern of genes regulating the HPA axis, namely *FKBP5*, *CRHR1*, and *NR3C1*, which are, however, not found in immune cells of the offspring. Increasing levels of maltreatment load were associated with lower mean methylation in the intron 7 of *FKBP5* and the *CRHR1* gene promoter, pointing towards a dose-dependent effect of CM. Furthermore, a higher mean methylation within exon 1 F of *NR3C1* was found in CM^+^ women compared to mothers without CM. Most importantly, the infants of CM^+^ mothers showed no differences in the methylation of *FKBP5*, *CRHR1*, and *NR3C1* compared to infants of CM^**−**^ mothers. Similarly, the gene expression levels of *NR3C1* and *FKBP5* were not correlated between mothers and infants, suggesting that there is no intergenerational transmission of methylation or gene expression patterns for these sites or transcripts in immune cells. The results provide first evidence that a history of CM does not affect immune cell methylation profiles of specific regulatory sites within stress-response-related genes in the offspring of maltreated mothers.

In line with previous studies^9–14^, our results show CM-associated hypomethylation of *FKBP5* and hypermethylation of *NR3C1*. We extend the existing literature by also showing effects on *CRHR1* methylation, thus providing a new candidate for CM-associated epigenetic alterations in HPA axis modulators. While the mean methylation levels of the three targeted genes differed in CM^+^ mothers compared to CM^**−**^ mothers, detailed analyses showed that these differences occur rather in specific CpG units. The observed mean methylation changes appeared not to be related to baseline gene expression alterations. Even though the sample size for gene expression analyses was smaller (47 mothers and 74 infant dropped out due to limited PBMC quantity) compared to the sample size for DNA methylation analyses, our results are in line with previous studies showing that hypomethylation of *FKBP5* intron 7 is associated with increased *FKBP5* gene expression *in vitro*, but only in response to dexamethasone stimulation and not under baseline conditions^14,29^. Moreover, even though the role of DNA methylation of stress-related genes in mental health outcomes has been repeatedly proposed^30–33^, it has not yet been shown whether this occurs via transcription changes of *FKBP5* and *NR3C1*. Our results show that CM-associated changes of *FKBP5* and *NR3C1* methylation are not necessarily associated to alterations in baseline expression of these genes. Regarding gene × environment interactions, our results further strengthen the perspective of an interactive effect of the *FKBP5* rs1360780 genotype and CM on *FKBP5* mean methylation^14,34^. Only T allele carriers showed a hypomethylation of *FKBP5* in association with CM, confirming previous results^13,14^. While the genotypic variation in *FKBP5* rs1360780 had no effect on *NR3C1* epigenetic regulation, it influenced the regulatory interrelation between *FKBP5* and *NR3C1*: We found a negative correlation between *FKBP5* and *NR3C1* methylation and a positive correlation between *FKBP5* and *NR3C1* gene expression only among the individuals with the CC genotype, but not among T carriers. Accordingly, previous studies showed that the stress response and recovery^35–37^ are rs1360780 genotype dependent; which, based on our results, could be a consequence of the dysregulation of the *FKBP5* and *NR3C1* interrelation.

The fact that the biomolecular regulatory changes were observed in immune cells suggests that environmental conditions during childhood might persistently (re)program central signaling cascades that influence immune functions. The observed epigenetic alterations of *FKBP5*, *CRHR1*, and *NR3C1* may translate to imbalanced immunology and thus provide a link between CM and the increased risk for somatic diseases in adulthood. Indeed, CM has been consistently associated with chronic low-grade inflammation^38,39^. A potential underlying mechanism is the CM-associated GR resistance phenotype and the consequently reduced cortisol-associated anti-inflammatory effect.

Investigating immune cells, we provide the first evidence that CM-associated epigenetic alterations in the selected genetic regions are not evident in the offspring. In this study, neonatal biological data were obtained from umbilical cord blood cells, allowing an intergenerational perspective that excludes the influence of parenting behaviors. The higher risk for immune-related conditions, such as asthma, allergies and auto-immune diseases described in the offspring of CM-exposed mothers compared to non-exposed mothers^23,40^ might be, thus, more related to CM-associated variations in mother-child interactions. These parenting interactions might result in higher stress levels, negatively impacting not only the child’s stress response regulation, but also development and health. In contrast to our observations, animal models have shown epigenetic changes in sperm cells in pups from early-life stressed^26,27^ and fear-conditioned^25^ mothers, which might be attributable to a tissue- or species-specificity of early life stress on DNA methylation patterns. Previous studies also described the importance of the intrauterine period in shaping the newborn’s development and DNA methylation patterns^24,41^. In particular, maternal stress exposure during pregnancy seems to be associated with specific DNA methylation changes in the *NR3C1* and *FKBP5* genes of the offspring, as shown in newborns^42–44^ and teenagers^45^. In our study, however, perceived maternal stress during the last 4 weeks prior to labor did not affect infants’ DNA methylation. Interestingly, grandmaternal interpersonal violence during pregnancy was recently found to be associated with altered DNA methylation patterns at specific CpG sites in their teenager grandchildren, highlighting the role of pregnancy stress on epigenetic regulation of the upcoming generations^46^. While the study, however, did not assess maternal methylation levels and multigenerational interactions, a generational gap of the transmission of CM-associated consequences could be hypothesized. In sum, our study design allows precluding the potentially biasing impact of the psychosocial mother-child interaction during the first years of life.

The following limitations need to be considered when interpreting our results: First, our study cohort comprised only mothers. When studying the intergenerational transmission of the effects of CM, the psychobiological relevance of the father also needs to be addressed. Indeed, research has shown differential effects of maternal and paternal diagnosis of posttraumatic stress disorder on their children’s *NR3C1* methylation^47^. Second, the epigenetic analyses were assessed in immune cells. Due to different biological material used across literature – most of them using whole blood for epigenetic analyses –, the comparability of our findings to those of other studies is difficult. It is unclear whether observations of environment-associated alterations of DNA methylation made in the periphery (immune cells) can be extrapolated to the central nervous system. New evidence suggests that an inter-tissue concordance between blood and brain cells is CpG specific^48^. However, PBMC are an accepted model for investigating the effects of chronic and traumatic stress on the body and previous work showed similar results with respect to CM-associated *NR3C1* hypermethylation in hippocampal cells from individuals who committed suicide^9^. Moreover, while our data suggest that the observed methylation changes of *FKBP5*, *CRHR1*, and *NR3C1* in PBMC from mothers with childhood maltreatment are not intergenerationally transmitted, these results cannot be generalized to other genomic sites and to other cells than immune cells. Thus, future investigations should investigate whether this finding can be extrapolated to other biological tissues and other gene loci. In addition, sample sizes differed among the different analyses. These differences were caused by the relatively stringent quality criteria we applied, the sample availability, and rs1360780 allelic distribution. Another limitation is that the specific immune subcell (e.g. B cells, T cells) distribution was not available. Based on studies that showed the importance of the blood cell type^49^, inclusion of specific subpopulation distributions of PBMC should be taken into account in future studies. Moreover, our sample included mothers in the perinatal period. Pregnancy, and especially delivery, can be considered as physiological stressors and cortisol levels are rising during the course of pregnancy, with a peak in the third trimester^50,51^. However, studies on CM-related DNA methylation alterations in non-pregnant cohorts showed similar results with regard to *NR3C1-1F* methylation^9,12,52^ and *FKBP5* methylation^13,14^, suggesting that these epigenetic alterations may be stable enough to persist under stressful circumstances (e.g., pregnancy and parturition).

In conclusion, this is the first study to assess methylation profile changes associated with CM in peripheral immune cells of both mothers and their offspring directly after birth. Our investigation of stress-response related genes that are pivotally involved in cortisol signaling cascades broadens the perspective of a long-lasting impact of CM on the interaction between the endocrine and the immune system, possibly affecting the duration and signaling of the stress response in the aftermath of CM. DNA methylation of the candidate genes *FKBP5*, *CRHR1*, and *NR3C1* in adaptive immune cells seems not to be intergenerationally transmitted from mothers with CM to their offspring. Although future studies are needed to confirm the results e.g. in an epigenome-wide approach, our results have an important implication: from the point of view of DNA methylation, the offspring of CM-exposed mothers does not seem to necessarily display the same epigenetic patterns as their mothers in the targeted CpG sites. In this case, professionals should focus on psychosocial factors during the first years of life, which might prospectively buffer the potential transmission of CM-associated consequences.

## Methods and Materials

### Study population and maternal CM exposure

Mother-infant dyads were recruited shortly after birth within the Department of Obstetrics and Gynecology of the Ulm University Hospital as part of the “My Childhood – Your Childhood” project. Umbilical cord blood was collected from infants born in the maternity ward of the Ulm University Hospital between October 2013 and December 2015 (*N* = 5426), transported to our laboratory and subjected to isolation of umbilical blood mononuclear cells (UBMC). Within one week after parturition, all mothers received a complete study description and were asked for participation. If the mothers did not fulfill any exclusion criteria (age under 18 years, insufficient knowledge of the German language, and severe health problems of mother or child during the course of pregnancy or labor) and gave written informed consent, venous blood drawing from the mothers was obtained. Infant UBMC were discarded immediately if mothers declined study participation. A total of 533 women provided written informed consent and basic socio-demographic data and completed a screening interview conducted by trained study personnel. The study was approved by the Ethics Committee of Ulm University and was conducted in accordance with the Declaration of Helsinki.

**Figure 5:**
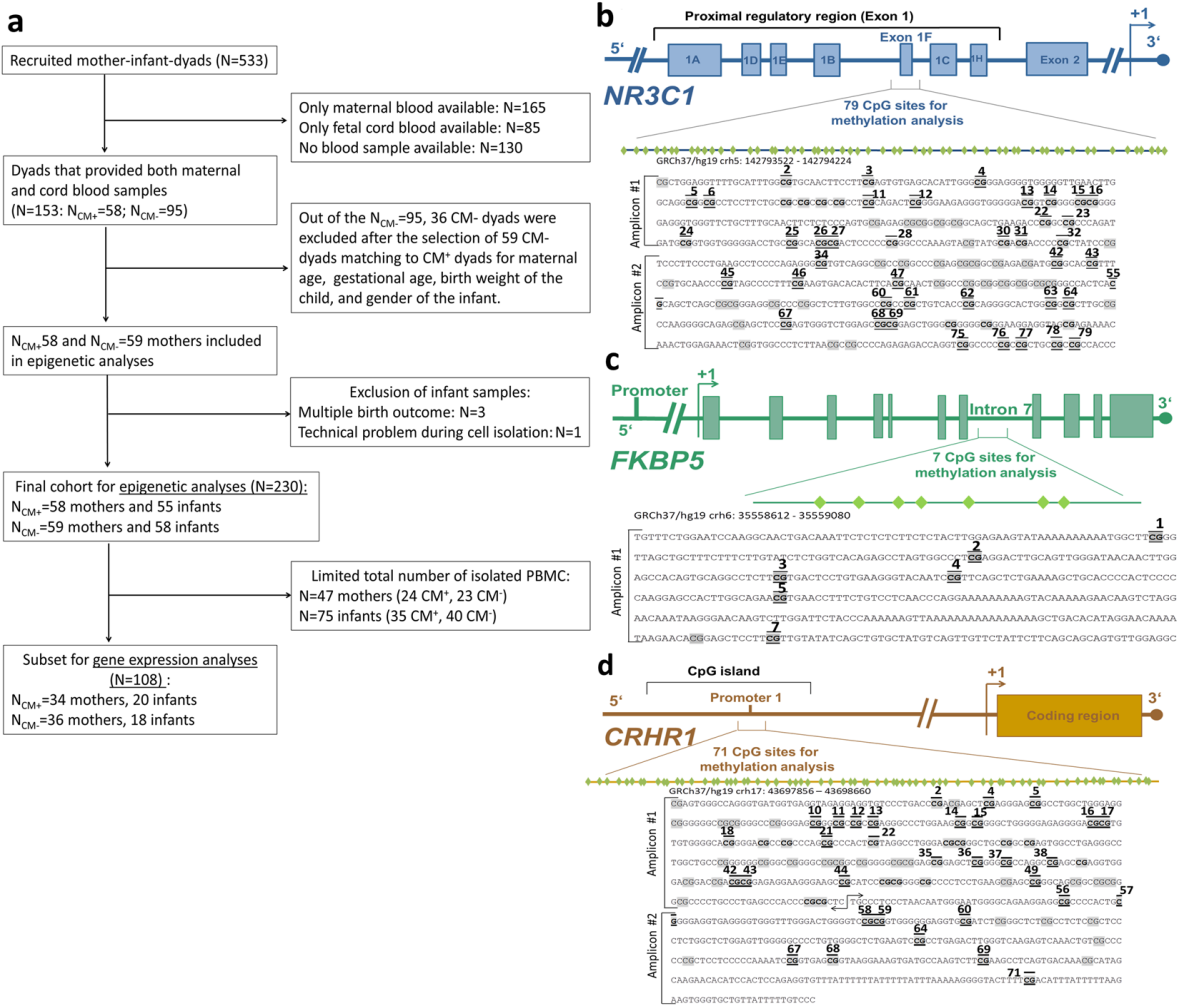
Schematic representation of the sample selection and the genetic targeted sequences. (**a**) Flowchart of inclusion criteria for epigenetic analyses. (**b**) For the *NR3C1* exon 1 F, 702 base pairs (bp) were included for the DNA methylation analyses that were analyzed through two amplicons (see Table S1 in the SI for primer information). (**c**) The intron 7 of the *FKBP5* genetic sequence has been especially under focus because it contains several GREs that potentially act as glucocorticoid-induced points for transcription start^14^. For the *FKBP5* intron 7, one single amplicon with 468 bp and 7 CpG sites was analyzed. (**d**) Two amplicons which covered a promoter region with a total length of 804 bp were analyzed for the *CRHR1* gene. All CpG sites included within the targeted sequence are highlighted in grey. CpG sites marked in bold generated a value by the mass spectrometry after high or low mass automatic discrimination. Underlined and up-lined are the CpG sites that remained for maternal and neonatal epigenetic analyses after the data preprocessing, respectively. Genomic and CpG islands annotations were based on the human UCSC Genome Browser (Feb. 2009, GRCh37/hg19) assembly.

The history of CM experiences was assessed with the German short version of the *Childhood Trauma Questionnaire* (CTQ)^53^. Mothers with mild to severe experiences in at least one subscale of the CTQ (emotional, physical or sexual abuse, and emotional or physical neglect) were categorized as CM^+^, elsewise as CM^**−**^ (according to the cut-offs described in^54^). Because of sample availability reasons, epigenetic analyses were conducted in a selected subset of study participants (see Fig. 5a and Supplemental Information (SI), section 1, for detailed information). The final cohort consisted of 117 mothers and 113 infants, with a maternal CTQ sum score between 25 and 85 (M ± SD = 33.6 ± 10.8).

### Isolation of immune cells and DNA isolation

Maternal peripheral blood and fetal umbilical cord blood were collected into CPDA-buffered collection tubes (Sarstedt S-Monovette, Nürmbrecht, Germany). Peripheral blood mononuclear cells (PBMC) from mothers and UBMC from infants were isolated by Ficoll-Hypaque density gradient centrifugation (GE Healthcare, Chalfon St Giles, UK) according to the manufacturer’s protocol. Cell pellets were resuspended into cryopreservation medium (dimethyl sulphoxide: Sigma-Aldrich, St. Louis, MO, USA; fetal calf serum: Sigma-Aldrich; dilution: 1:10) and stored at −80 °C until DNA isolation. An additional sample of whole blood collected into EDTA-buffered blood collection tubes (Sarstedt S-Monovette) was available from *N* = 108 mothers and *N* = 90 infants, which was used for standard blood cell counts at the Department of Clinical Chemistry and Central Laboratory of Ulm University. For the epigenetic and genotyping analyses, genomic DNA from PBMC and UBMC was isolated using the MagNaPure 96 system (Roche, Basel, Switzerland). DNA concentrations were quantified using a Qubit spectrophotometer (Life Technologies, Carlsbad, CA, USA). DNA was lyophilized in a CentriVap concentrator (Labconco Corp, MO, USA) and resuspended in DNAse-free water (Life Technologies) to obtain a final DNA concentration of approximately 40 ng/µl. Frozen DNA samples were handed over to Varionostic GmbH (Ulm, Germany) for mass array methylation analyses.

### DNA methylation analyses

For *NR3C1* and *FKBP5*, the targeted genomic regions for methylation analyses were previously reported as regulatory areas, namely the intron 7 within *FKBP5*^14^ and exon 1F of *NR3C1*^9,44^. For the analyses of the *CRHR1* gene, a CpG island (a genomic region rich on cytosine-guanine-dinucleotide sequences) in a known promoter region, 84 bp downstream from a transcription start point (Fig. 5b–d), was targeted for DNA methylation analyses.

After bisulfite treatment of 500 to 1000 ng of genomic DNA, PCR-based amplification, reverse transcription and complete enzymatic cleavage with RNase A, mass spectrometry was performed according to the EpiTYPER protocol, which is technically based on Matrix-Assisted Laser Desorption/Ionization Time-of-Flight (MALDI-TOF) Mass Spectrometry (Sequenom Inc, San Diego, CA, USA). For detailed information on the targeted CpG sites see SI, section 2. Samples were measured blinded to the experimenter.

### Gene expression analyses

Gene expression analyses were conducted in a subsample of *N* = 70 mothers and *N* = 39 infants because of sample availability reasons (see SI, Section 1 for further details). RNA was purified using the Qiagen RNeasy Kit (QIAGEN, Hilden, Germany) and quantified with a Qubit spectrophotometer (Life Technologies). RNA was stored in *RNase* free water (Life Technologies) at −20 °C for up to seven days prior to cDNA transcription using a high-capacity cDNA reverse transcription Kit (Thermo Fischer Scientific, Darmstadt, Germany) following the manufacturer’s instructions. The assessment of gene expression by Real-time qPCR analyses was performed on a QuantStudio 6 (Life technologies) with TaqMan gene expression arrays (Thermo Fischer Scientific): Hs01561006_m1 for *FKBP5* and Hs00353740_m1 for *NR3C1*. *CRHR1* gene expression was not detected in PBMC and UBMC (see SI, section 3, for further information). Two internal controls were used as reference genes: succinate dehydrogenase complex, subunit A (*SDHA*; Hs00188166_m1) and Importin 8 (*IPO8*; Hs00183533_m1; SI, Section 4). Reactions containing 20 ng of cDNA in a total volume of 20 µl were performed in triplicates and separately for each target gene. After calculating the average Ct of the measured triplicates, the relative mRNA levels of *FKBP5* and *NR3C1* were defined with the 2^−∆Ct^ equation, with ∆Ct = (mean Ct of the target) − (geometric mean of the Ct of the reference genes *SDHA* and *IPO8*). The resulting fold-change values – an estimate of relative mRNA expression levels – were used for statistical analyses.

### *FKBP5* rs1360780 genotyping

The rs1360780 genotype was assessed in a LightCycler® 480 (Roche Applied Science, Penzberg, Germany) using the Roche HybProbe system and melting curve technology (see SI, section 5 for more information). The minor allele (T) frequency (MAF) was 23.5% in mothers and 26% in infants. The rs1360780 genotype frequencies were in Hardy-Weinberg equilibrium (*χ*^2^_(2)_ = 1.23, *p* = 0.27) and the T allele frequencies here described are comparable to previous studies^13,55,56^. Since the frequency of the homozygote TT genotype was relatively low (7.7% in mothers and 7.1% in infants), we dichotomized samples into “carriers” of the T allele (genotypes CT and TT) and “non-carriers” (CC), as reported in 14.

### Data pre-processing and statistical analyses

Data processing and statistical analyses were conducted with R version 3.2.3^57^. In order to pre-process the methylation data generated by the spectrometry, we applied two quality criteria to ensure high quality of the raw data (see SI, section 6). For each candidate gene, the mean percentage of methylation over all the CpG sites was calculated and used for statistical analyses.

Student *t*-tests and χ^2^*-*tests were used for demographic and clinical descriptive analyses. Normal distribution of the model residuals was tested with the *Shapiro-Wilk* test. All methylation data was skewed and thus the non-parametric *Wilcoxon* tests and *Kendall’s tau* correlations were used for group comparisons and correlation analyses, respectively. We further included potentially influencing factors as covariates: maternal age, reported maternal glucocorticoid medication intake (e.g. corticosteroid sprays or topical creams), and time from delivery until PBMC isolation in days when analyzing maternal data, and gestational age at birth and gender for the tests with infant’s data. Preparatory analyses showed no significant group differences in the relative amount of monocytes and lymphocytes between CM^+^ and CM^−^ women (Table 1). Cell counts did not show a significant correlation with the methylation levels for any of the epigenetic sites and were thus not included as covariates. Since the assumptions for statistical testing under application of linear models were violated (e.g. not normally distributed residuals), non-parametric permutation tests^58^ were used to test for statistical effects of the covariates or interaction analyses. A detailed description of the permutation tests can be found in the SI (section 7). Based on previous findings of a *FKBP5* × CM interaction on *FKBP5* methylation^14^ and of hypomethylation of *FKBP5*^13,14^ and hypermethylation of *NR3C1*^9–13^ in association with CM, directed one-tailed tests were conducted for *FKBP5* and *NR3C1* mean methylation analyses. Statistical analyses for *CRHR1* and all other tests were performed with two-tailed tests. To counteract the risk of false positives, the False Discovery Rate adjustment (FDR^59^) was used for individual CpG analyses (SI, section 8). The level of significance was set at *p* ≤ 0.05.

## Electronic supplementary material

Supplementary Information

## Data Availability

The anonymized datasets generated during and/or analyzed during the current study are available from the corresponding author upon reasonable request.
