# Supplementary material for: The effects of childhood maltreatment on epigenetic regulation of stress-response associated genes: an intergenerational approach
Source: Sci Rep. 2019 Apr 18;9:983. doi: 10.1038/s41598-018-36689-2 (PMC7052131; doi:10.1038/s41598-018-36689-2)
Supplement: Supplementary file 1 — Supplementary Information [file 41598_2018_36689_MOESM1_ESM.docx]

**The effects of childhood maltreatment on epigenetic regulation of stress-response associated genes: an intergenerational approach**

Supplementary Information

Laura Ramo-Fernández^1^, Christina Boeck^1^, Alexandra M. Koenig^1^, Katharina Schury^1,2^, Elisabeth B. Binder^3,4^, Harald Gündel^5^, Jörg M. Fegert^2^, Alexander Karabatsiakis^1^, Iris-Tatjana Kolassa^1*^.

^1^ Clinical & Biological Psychology, Institute of Psychology & Education, Ulm University, Ulm, 89081, Germany.

^2^ Department of Child and Adolescent Psychiatry and Psychotherapy, University Hospital Ulm, 89075, Ulm, Germany.

^3^ Department of Translational Research in Psychiatry, Max Planck Institute of Psychiatry, Munich, 80804, Germany.

^4^ Department of Psychiatry and Behavioral Sciences, Emory University School of Medicine, Atlanta, GA 30322, USA.

^5^ Department of Psychosomatic Medicine and Psychotherapy, University Hospital Ulm, 89081, Ulm, Germany.

1. *Study participants included for epigenetic and gene expression analyses*

A total of *N=*533 of women provided written informed consent for study participation. Out of all dyads that provided both maternal blood and fetal cord blood samples (*N=*153), all 58 pairs of CM^+^ women and their infants were included in epigenetic analyses. With the focus on those mothers who were willing to further participate in the overall project (mothers-infant-dyads were invited to participate in two more psycho-diagnostic interviews: 3 months and 1 year after birth) and matching for maternal age, gestational week at the time of birth, birth weight, and gender of the infants, 59 CM^-^ mother-infant-dyads were selected for epigenetic analyses as controls (CM^-^). CM^-^ mothers that were included in the epigenetic analyses did not differ from CM^-^ mothers that were not included (*N=*36) with respect to maternal age, CTQ sum score, gender, and weight of their newborns, ethnicity, cigarette smoking habits during pregnancy, chronic illnesses, lifetime psychological diagnosis or medication during pregnancy (all *p*-values>.05). Similarly, mothers who were included for epigenetic analyses (*N=*117) did not differ from mothers from the larger sample who were not included (*N=*416) in terms of maternal chronological age, gender and body weight of their newborns, ethnicity, cigarette smoking during pregnancy, chronic illnesses, lifetime psychological diagnosis or medication intake during pregnancy (all *p*-values >.05). After excluding the offspring of three mothers who gave birth to twins, the cohort included 114 infants. The cells from one of these infants were not available due to technical problems during cell isolation. Thus, the final cohort included 117 mothers and 113 infants. Clinical characteristics and medication during pregnancy of the included mothers are described in Table S1. Regarding the samples included for gene expression analyses, RNA could be extracted from 70 mothers and 39 infants, since for some participants (*N=*47 mothers, *N=*74 infants) the total number of isolated PBMC was limited, and therefore not available for RNA isolation (Fig 1 in main text). After having performed *FKBP5* gene expression analyses, there was no remaining cDNA left for gene expression analyses for *NR3C1* from one mother and from two children. *FKBP5* gene expression analysis from one of the children was missing because of technical failure during qPCR performance.

1. *Targeted CpG sites*

Genomic and CpG island annotations were based on the human UCSC Genome Browser (Feb. 2009, GRCh37/hg19) assembly. The targeted areas encompassed the following specific genomic regions: *FKBP5*, GRCh37/hg19 crh6: 35558612 – 35559080; *CRHR1*, GRCh37/hg19 crh17: 43697856 – 43698660 and *NR3C1*, GRCh37/hg19 crh5: 142793522 – 142794224. For *FKBP5,* 7 CpG sites were analyzed. For the targeted *CRHR1* region, the EpiTYPER yielded 40 CpG units that included between one and eight CpG sites, and for *NR3C1* exon 1F analyses, 40 CpG units that included between one and seven CpG sites. Those DNA fragments that had low or high mass outside of the detection range of the Sequenom EpiTYPER were set as missing data (1 CpG unit in *FKBP5*, 11 CpG units in *CRHR1,* 10 CpG units in *NR3C1*). The primers that were employed for mass array-based DNA methylation analyses are described in Table S2. The special nature of the EpiTYPER-based technique does not allow the discrimination between DNA fragments with identical molecular weight and thus these cannot be measured independently (e.g.: CpG 2.3.34 from *NR3C1*).

1. *CRHR1 gene expression test in PBMC*

Since no study so far reported expression of *CRHR1* in human PBMC, we initially tested *CRHR1* expression (Taqman gene expression array Hs00366363_m1; Thermo Fischer Scientific) in PBMC together with selected commercially available human carcinogenic cell lines that served as positive controls (A549, human adenocarcinomic alveolar basal epithelial cells; LS174T, colon cancer human cells). While A549 and LS174T showed a fluorescent signal for *CRHR1*, no fluorescent signal was observed in PBMC, indicating no detectable baseline expression of CRHR1 in human PBMC. Thus, *CRHR1* gene expression was not conducted.

1. *Selection of SDHA and IPO8 as RT-PCR reference controls*

The two housekeeping genes succinate dehydrogenase complex, subunit A (*SDHA*) and Importin 8 (*IPO8*) were used as reference genes. They were pre-selected out of a total of five candidates genes (*SDHA, IPO8, 18S, TBP*, *RPL13A*) that were reported in the literature to be stably expressed in human PBMC (1). Using the program NormFinder (2), an algorithm-based test that identifies the optimal normalization gene among the set of candidates, we ranked the candidates genes according to their expression stability in the given sample set and experimental design. Based on this algorithm, the combination of *IPO8* and *SDHA* generated the strongest stability and were thus selected as reference genes. Both housekeeping genes were equally expressed in CM^-^ and CM^+^ groups (*t_SDHA(1,64)_=-*0.93, *p*=.35; *t_IPO8(1,66)_=*0.13, *p=.*90).

1. *FKBP5 rs1360780 SNP genotyping conditions*

For the genotyping of *FKBP5* rs1360780, the LightCycler® 480 (Roche Applied Science, Penzberg, Germany) was employed. This real-time PCR technique allows genotyping assessment based upon the different melting temperatures for the two SNP alleles C (67 °C) and T (71°C). The Roche genotyping Mastermix and the following primers were employed for rs1360780 genotyping: forward primer: CCTTATTCTATAGCTGCAAGTCCC; reverse primer: TCTGAATATTACCAGGATGCTGAG; sensor probe: CAGAAGGCTTTCACATAAGCAAAGTTACACAAAAC-Fluorescein and anchor probe: LC Red 640-AAATTCTTACTTGCTACTGCTGGCACAAGAGA-Phosphate. The amplification protocol consisted of 45 cycles with 10 seconds each at 95 °C, 56 °C and then 72 °C followed by a melting curve analysis. Positive and negative controls were used for additional quality control.

1. *Pre-processing of the DNA methylation data*

In order to pre-process the methylation raw data for analyses, the following quality criteria were applied for each gene: First, CpG units with more than 30% of missing values in the raw data from the mass spectrometry were excluded from all analyses (3,4). This step was applied for all mothers and all infants separately. Six CpG sites remained for *FKBP5* in mothers and infants, 27 CpG sites for *CRHR1* in mothers (32 CpG sites in infants) and 29 CpG sites remained for *NR3C1* analyses in mothers (41 CpG sites in infants). For a graphical representation of the included CpG units within each gene see Fig. 2. A second quality criterion was the exclusion of the data from individuals with more than 50% of missing values across all CpG units of each targeted gene. The final dataset consisted of 109 mothers and 112 infants for *FKBP5*, 114 mothers and 109 infants for *CRHR1,* and 113 mothers and 101 infants for *NR3C1*.

1. *Description of the permutation tests*

In case of not normally distributed model residuals, non-parametric permutation tests (5) were applied as they are rather robust to the violations of ordinary least square (OLS) model assumptions (i.e. normality of model residuals). We used permutation under the reduced model, i.e., a model reduced by the variable of interest was fitted, residuals were permuted randomly, the full model was fitted and the *t* statistic *t’* was recorded. This *t’* statistic was iterated 10,000 times. Finally, the *t* value obtained from the original data was compared to the distribution of the 10,000 *t’* values obtained by the permutation test, and the *p* value was determined as the proportion of *t’* values exceeding *t*, respectively (6). Standardized *β* coefficients and permutation-based *p* values are reported.

1. *Specific CpG*  units analyses

Since literature suggests that specific CpG units can be hyper- or hypo-methylated independently of the direction of the average methylation of the region, we decided to report all *p*-values as result of two-sided tests from single unit analyses. The detailed analyses of the association between DNA methylation and CM in the specific CpG units showed that not all CpG units but rather single units show group differences in mothers (Table S4). Within the *FKBP5* targeted genetic region, CpG 2, CpG 4, CpG 5 and CpG 7 were significantly less methylated in CM^+^ mothers, while the CpG 3 was higher methylated in CM^+^ women compared to CM^-^ women (cf. 7). Regarding *CRHR1*, only the CpG 60 was higher methylated in CM^+^ compared to CM^-^ women. With respect to *NR3C1*, the unit CpG 4.12 was significantly lower methylated in CM^+^, while the CpG 5.6 and CpG 25.26.27 were higher methylated in CM^+^ mothers compared to CM^-^ women.

Reference list from Supplementary Information

1. Ledderose, C., Heyn, J., Limbeck, E. & Kreth, S. Selection of reliable reference genes for quantitative real-time PCR in human T cells and neutrophils. *BMC Res. Notes* **4**, 427 doi: 10.1186/1756-0500-4-427 (2011).
2. Andersen, C. L., Jensen, J. L. & Ørntoft, T. F. Normalization of real-time quantitative reverse transcription-PCR data: a model-based variance estimation approach to identify genes suited for normalization, applied to bladder and colon cancer data sets. *Cancer Res.* **64**; 5245-5250 (2004).
3. Steiger, H., Labonte, B., Groleau, P., Turecki, G. & Israel, M. Methylation of the glucocorticoid receptor gene promoter in bulimic women: associations with borderline personality disorder, suicidality, and exposure to childhood abuse. *Int. J. Eat Disord.* **46**; 246-255 (2013).
4. Mansell, T., *et al*. Maternal mental well-being during pregnancy and glucocorticoid receptor gene promoter methylation in the neonate. *Dev. Psychopathol.* **28**; 1421-1430 (2016).
5. Freedman, D. & Lane, D. A nonstochastic interpretation of reported significance levels. *J. Bus. Econ. Stat.* **1**; 292-298 (1983).
6. Anderson, M. J. & Robinson, J. Permutation tests for linear models. *Aust. N. Z. J. Stat.* **43**; 75-88 (2011).
7. Klengel, T., *et al.* Allele specific FKBP5 DNA demethylation mediates gene–childhood trauma interactions. *Nat. Neurosci.* **16**; 33–41 (2013).

| **Table S1.** Clinical characteristics and medication during pregnancy *^a^* | | | | | | | | |  |  |  |
| --- | --- | --- | --- | --- | --- | --- | --- | --- | --- | --- | --- |
|  |  |  |  |  | CM^-^ | CM^+^ |  |  |  |  |  |
|  |  |  |  |  | (N=59) | (N=58) | Statistics | *p*^b^ |  |  |  |
| Self-reported psychiatric diagnosis (lifetime) | | | | | |  |  |  |  |  |  |
|  | Lifetime diagnosis *(N (%))* | | | | 12 (20.3) | 17 (29.3) | *χ^2^ _(1)_=*0.83 | .36 |  |  |  |
|  | Depressive disorder *(N (%)^c^* | | | | 7 (11.9) | 5 (8.8) | *χ^2^ _(1)_=*0.78 | .38 |  |  |  |
|  | Anxiety disorder *(N (%))*^d,e^ | | |  | 2 (3.6) | 5 (8.8) | *χ^2^ _(1)_=*0.12 | .73 |  |  |  |
|  | Eating disorder *(N (%))* | | |  | 0 | 2 (3.4) | *χ^2^ _(1)_=*0.53 | .47 |  |  |  |
|  | Adjustment disorder (*N (%))* | | |  | 0 | 2 (3.4) | *χ^2^ _(1)_=*0.53 | .47 |  |  |  |
|  | Other psychiatric diagnoses *(N (%))* | | | | 3 (5.1) | 3 (5.2) | *χ^2^ _(1)_=*0.19 | .66 |  |  |  |
| Chronic illnesses^f^ | | |  |  |  |  |  |  |  |  |  |
|  | Thyroid dysfunction *(N (%))* | | |  | 11 (18.6) | 8 (13.8) | *χ^2^ _(1)_=*0.21 | .65 |  |  |  |
|  | Allergy *(N (%))* | |  |  | 10 (16.9) | 7 (12.1) | *χ^2^ _(1)_=*0.24 | .63 |  |  |  |
|  | Neurodermatitis *(N (%))* | | |  | 2 (3.4) | 1 (1.7) | *χ^2^ _(1)_<*0.001 | 1 |  |  |  |
|  | Diabetes (*N (%)*) | |  |  | 2 (3.4) | 1 (1.7) | *χ^2^ _(1)_<*0.001 | 1 |  |  |  |
| Medication during pregnancy | | | |  |  |  |  |  |  |  |  |
|  | L-Thyroxin *(N* (%)) | |  |  | 14 (23.7) | 10 (17.2) | *χ^2^ _(1)_=*0.41 | .52 |  |  |  |
|  | Glucocorticoids (*N (%)*) | | |  | 2 (3.4) | 3 (5.2) | *χ^2^_(1)_<*0.001 | 1 |  |  |  |
|  | Anti-depressants (*N* (%)) | | |  | 0 | 1 (1.7) | *χ^2^ _(1)_<* 0.001 | .99 |  |  |  |
| Women with at least mild CM experiences in one subscale of the CTQ were categorized as CM^+^, otherwise as CM^-^. CM= Childhood maltreatment; CTQ= *Childhood Trauma Questionnaire*. | | | | | | | | |  |  |  |
| ^a^ Disease and medication were listed when at least 2 individuals had the diagnosis or were medicated | | | | | | | | | | | |
| ^b^ Main effect of the CM classification (chi-square tests). | | | | | |  |  |  |  |  |  |
| ^c^ Data regarding lifetime diagnosis of depression was not available for one CM^+^ women. | | | | | | | | |  |  |  |
| ^d^ One woman from each CM group had a diagnosis of depression and anxiety disorder. | | | | | | | | |  |  |  |
| ^e^ Data regarding lifetime diagnosis of anxiety disorder was not available for one woman of the CM^+^ group. | | | | | | | | |  |  |  |
| ^f^ One CM+ women had asthma, neurodermatitis, and allergy; one CM+ had diabetes and thyroid dysfunction; one CM+ woman had an allergy and thyroid dysfunction. | | | | | | | | |  |  |  |

| **Table S2**. Sequencing primers for EpiTYPER analyses | | |
| --- | --- | --- |
| Gene symbol | Amplicon | Sequencing primer (5'-3')^a^ |
| *NR3C1* | *NR3C1* Exon 1F #1 | F: aggaagagagGTTTTTTGGGGAGGTTTTAGGG |
|  |  | R:cagtaatacgactcactatagggagaaggctAAAAAAATACAAAAAAATCCAACTC |
|  | *NR3C1* Exon 1F #2 | F: aggaagagagTTTAATTTTTTAGGAAAAAGGGTGG |
|  |  | R:cagtaatacgactcactatagggagaaggctCCCTAAAACCTCCCCAAAAAAC |
| *FKBP5* | *FKBP5* Intron 7 #1 | F: aggaagagagGTTGTTTTTGGAATTTAAGGTAATTG |
|  |  | R: cagtaatacgactcactatagggagaaggct TCTCTTACCTCCAACACTACTACTAAAA |
| *CRHR1* | *CRHR1* #1 | F: aggaagagagGTTGTTTTTGGTATTGGTGTTTTTG |
|  |  | R:cagtaatacgactcactatagggagaaggctCTTCTACCCCATTCCCATTATTAAA |
|  | *CRHR1* #2 | F: aggaagagagTTTTTTTAATAATGGGAATGGGGTA |
|  |  | R:cagtaatacgactcactatagggagaaggctAAAACAAAAATAACAACACCCACTT |
| ^a^ Capital letters: gene specific | |  |

| **Table S3**. Gene expression analyses | | | |  |  |  |  |
| --- | --- | --- | --- | --- | --- | --- | --- |
|  | Target | Total (*n=*70) | | CM^-^ (*n=*36) | CM^+^ (*n=*34) | Statistics | *p^a^* |
| Mothers |  |  | |  |  |  |  |
|  | *FKBP5 (M*(*SD))* | 2.11 (0.92) | | 2.04 (0.75) | 2.20 (1.07) | *W*= 671 | .49 |
|  | *NR3C1^b^  (M*(*SD))* | 1.43 (0.62) | | 1.60 (0.99) | 1.39 (0.50) | *W=* 629 | .69 |
| Newborns | |  | |  |  |  |  |
|  | *FKBP5^c^ (M*(*SD))* | 3.62 (2.14) | | 3.61 (2.54) | 3.62 (1.76) | *W=* 158 | .71 |
|  | *NR3C1^d^ (M*(*SD))* | 2.54 (0.89 | | 2.53 (0.88) | 2.82 (1.52) | *W=* 157 | .71 |
| M= mean of fold change, relative mRNA levels. | | |  |  |  |  |  |
| *^a^* Statistics: Wilcox-test when data was not normally distributed. Main effect of the CTQ classification. | | | | | | | |
| ^b^ CM^-^: *N=* 35 | |  | |  |  |  |  |
| ^c^ CM^-^: *N=* 18, CM^+^: *N=* 19 | |  | |  |  |  |  |
| ^d^ CM^-^: *N=* 17, CM^+^: *N=* 20 | |  | |  |  |  |  |

| **Table S4.** Group-wise specific CpG unit analyses in mothers and infants | | | | | | | |  |  |  |  |  |  | |  |  |
| --- | --- | --- | --- | --- | --- | --- | --- | --- | --- | --- | --- | --- | --- | --- | --- | --- |
|  |  | **Mothers** | | | | | |  |  | **Infants** | | | | | |  |
|  |  |  | **CM^-^** | **CM^+^** |  | |  |  |  | **CM^-^** | **CM^+^** |  | |  |  |  |
| **Gene** | **CpG unit** | **N** | **Mean % methylation (SD)** | **Mean % methylation (SD)** | **Statistics** | ***p-*value FDR adjusted** | |  | **N** | **Mean % methylation (SD)** | **Mean % methylation (SD)** | **Statistics** | | ***p-*value FDR adjusted** |  |  |
| ***FKBP5*** | CpG 1 | 109 | 68.9 (34.6) | 70.3 (24.2) | *W=*1707 | | .18 |  | 111 | 66.3 (25.3) | 63.7 (27.3) | *W=*1641 | | .96 |  |  |
|  | CpG 2 | 102 | 89.1 (10.7) | 78.4 (9.2) | *W=*2093.5 | | **<.0001** |  | 111 | 91.3 (8.8) | 90.4 (12.2) | *W*=1511.5 | | .96 |  |  |
|  | CpG 3 | 109 | 75.9 (15.9) | 81.5 (12.2) | *W=*978 | | **<.01** |  | 111 | 79.2 (12.2) | 81.1 (8.3) | *W=*1379 | | .96 |  |  |
|  | CpG 4 | 104 | 80.0 (14.8) | 73.8 (6.6) | *W*=2149 | | **<.0001** |  | 109 | 78.2 (8.3) | 79.1 (7.8) | *W=*1463 | | .96 |  |  |
|  | CpG 5 | 96 | 82.0 (20.9) | 73.8 (10.4) | *W*=1736.5 | | **<.0001** |  | 101 | 79.4 (14.6) | 79.2 (16.7) | *W*=1253.5 | | .96 |  |  |
|  | CpG 7 | 105 | 84.8 (11.5) | 69.6 (10.9) | *t(103)*=6.94 | | **<.0001** |  | 111 | 89.8 (5.9) | 90.2 (5.9) | *W=*1531 | | .96 |  |  |
| ***CRHR1*** | CpG 2^a^ | - | - | - | - | | - |  | 84 | 10.7 (9.1) | 12.9 (10.7) | *W=*793 | | .64 |  |  |
|  | CpG 4 | 83 | 0.8(1.9) | 0.9 (2) | *W=*776 | | .60 |  | 92 | 3.3 (3.8) | 2.1 (2.9) | *W=*1724 | | .45 |  |  |
|  | CpG 5 | 83 | 3.8 (1.5) | 3.8 (1.0) | *W=*885.5 | | .87 |  | 92 | 4.6 (1.6) | 4.4 (1.6) | *W=*1131 | | .72 |  |  |
|  | CpG 10.11.12.13 | 83 | 1.9 (2.9) | 3.1 (5.6) | *W=*681.5 | | .34 |  | 92 | 2.1 (1.8) | 1.9 (2.1) | *W=*1165 | | .64 |  |  |
|  | CpG 14.15 | 83 | 7.4 (2.5) | 7 (2.4) | *W=*858.5 | | .98 |  | 92 | 8.2 (2.2) | 7.8 (2.1) | *t(90)*=0.98 | | .60 |  |  |
|  | CpG 16.17 | 83 | 9.5 (3.2) | 9.3 (3.8) | *t(81)*=0.29 | | .77 |  | 92 | 1.8 (1.1) | 1.6 (1.2) | *W=*1624 | | .64 |  |  |
|  | CpG 18^a^ | - | - | - | - | | - |  | 92 | 13.5 (7.2) | 13.1 (9.1) | *W=*1038 | | .78 |  |  |
|  | CpG 21 | 83 | 1.0 (2.6) | 0.4 (0.9) | *W=*873 | | .88 |  | 92 | 1.0 (2.0) | 1.2 (1.5) | *W=*897 | | .46 |  |  |
|  | CpG 22^a^ | - | - | - | - | | - |  | 81 | 12.9 (7.2) | 17.8 (10.3) | *W=*526.5 | | .15 |  |  |
|  | CpG 35^a^ | - | - | - | - | | - |  | 87 | 13.1 (10.2) | 15.8 (10.0) | *W=*774 | | .45 |  |  |
|  | CpG 36.37 | 83 | 3.2 (4.0) | 3.1(4.1) | *W=*898 | | .82 |  | 92 | 3.5 (2.8) | 3.6 (4.2) | *W=*1174.5 | | .64 |  |  |
|  | CpG 38 | 83 | 6.6 (5.0) | 8.1 (5.7) | *W=*737.5 | | .60 |  | 92 | 6.0 (5.3) | 7.6 (4.7) | *W=*743.5 | | .18 |  |  |
|  | CpG 42.43 | 83 | 27.0 (11.35) | 29.4 (12.9) | *W=*792.5 | | .77 |  | 92 | 24.0 (11.01) | 26.3 (11.7) | *W=*1247 | | .64 |  |  |
|  | CpG 44 | 83 | 0.8(1.9) | 0.9 (2) | *W=*776 | | .60 |  | 92 | 3.3 (3.8) | 2.1 (2.9) | *W=*1131 | | .45 |  |  |
|  | CpG 49 | 83 | 3.7(2.2) | 3.5 (2.4) | *W=*903.5 | | .82 |  | 92 | 4.3 (2.2) | 4.1 (2.4) | *W=*1165 | | .64 |  |  |
|  | CpG 56 | 108 | 0.9 (0.1) | 1.2 (0.9) | *W=*1222 | | .35 |  | 109 | 1.9 (1.1) | 1.6 (1.2) | *W=*1724 | | .15 |  |  |
|  | CpG 57 | 85 | 1.7 (1.4) | 1.9 (1.3) | *W=*756 | | .62 |  | 109 | 2.6 (1.9) | 2.5 (1.9) | *W*=1508 | | .82 |  |  |
|  | CpG 58.59 | 107 | 6.8 (2.8) | 7.2 (2.4) | *W=*1228.5 | | .49 |  | 100 | 10.5 (7.0) | 9.4 (7.3) | *W=*1542 | | .46 |  |  |
|  | CpG 60 | 108 | 0.8 (1.1) | 1.3 (1.2) | *W=* 991.5 | | **.047** |  | 109 | 1.7 (1.2) | 1.8 (1.2) | *W*= 1394 | | .63 |  |  |
|  | CpG 64 | 85 | 0.6 (0.9) | 1.1 (1.0) | *W=* 595 | | .13 |  | 109 | 1.8 (1.1) | 1.6 (1.2) | *W=* 1624 | | .37 |  |  |
|  | CpG 67 | 107 | 13.8 (13.2) | 5.3 (5.5) | *W=* 1801 | | .13 |  | 109 | 3.9 (1.2) | 3.6 (1.3) | *W=* 1766,5 | | .14 |  |  |
|  | CpG 68^a^ | - | - | - | - | | - |  | 90 | 4.7 (5.3) | 4.8 (6.1) | *W=* 1054 | | .93 |  |  |
|  | CpG 69 | 84 | 1.4 (1.4) | 2.4 (2.4) | *W=* 660 | | .33 |  | 109 | 3.3 (2.1) | 3.1 (1.9) | *W=* 1575,5 | | .49 |  |  |
|  | CpG 71 | 85 | 10.8 (7.9) | 8.2 (4.5) | *W=* 1026 | | .34 |  | 109 | 7.7 (2.9) | 7.3 (2.9) | *W=* 1642 | | .51 |  |  |
| ***NR3C1*** | CpG 2.3.24 | - | - | - | - | | - |  | 88 | 17.2 (6.8) | 17.7 (6.6) | *t(86)*= -0.37 | | .90 |  |  |
|  | CpG 4.12 | 80 | 16.7 (4.9) | 13.4 (2.2) | *W=* 1137.5 | | **.001** |  | 94 | 17.2 (3.0) | 17.3 (3.0) | *W=* 1040.5 | | .84 |  |  |
|  | CpG 5.6 | 79 | 1.8 (2.0) | 2.4 (1.2) | *W=* 365 | | **.01** |  | 95 | 1.2 (1.0) | 1.9 (1.2) | *W=* 755.5 | | .09 |  |  |
|  | CpG 11 | 80 | 0.5 (1.2) | 0.5 (1.1) | *W=* 791 | | .96 |  | 95 | 0.6 (1.7) | 0.5 (1.0) | *W=* 1098 | | .88 |  |  |
|  | CpG 13 | 80 | 2.7 (1.0) | 3.0 (1.2) | *W=* 647 | | .46 |  | 95 | 3.0 (1.4) | 3.3 (0.9) | *W=* 915.5 | | .49 |  |  |
|  | CpG 14.15.16^a^ | - | - | - | - | | - |  | 83 | 4.3 (2.6) | 4.3 (2.7) | *W=* 834.5 | | .88 |  |  |
|  | CpG 22.23^a^ | - | - | - | - | | - |  | 93 | 2.1 (1.4) | 1.9 (1.4) | *W=* 1151 | | .84 |  |  |
|  | CpG 25.26.27 | 79 | 4.4 (3.3) | 6.5 (2.3) | *W=* 365 | | **.001** |  | 95 | 5.2 (2.0) | 5.4 (1.6) | *W=* 1113 | | .92 |  |  |
|  | CpG 28^a^ | - | - | - | - | | - |  | 92 | 4.9 (4.5) | 5.2 (4.4) | *W=* 1005.5 | | .84 |  |  |
|  | CpG 30.31.32^a^ | - | - | - | - | | - |  | 93 | 3.8 (2.6) | 4.2 (2.0) | *W=* 887.5 | | .53 |  |  |
|  | CpG 34 | 113 | 0.7 (0.8) | 1.2 (3.3) | *W=* 1524.5 | | .96 |  | 99 | 0.7 (0.9) | 1.2 (1.1) | *W=* 908 | | .14 |  |  |
|  | CpG 42.43 | 113 | 1.4 (0.8) | 1.6 (0.8) | *W=* 1358 | | .32 |  | 99 | 1.7 (1.0) | 1.7 (0.9) | *W=* 1316 | | .84 |  |  |
|  | CpG 45 | 113 | 10.8 (4.0) | 10.9 (3.4) | *W=* 1597 | | .99 |  | 99 | 11.6 (2.5) | 10.6 (3.0) | *W=* 1397 | | .77 |  |  |
|  | CpG 46 | 108 | 11.7 (7.1) | 11.8 (7.5) | *W=* 1435.5 | | .99 |  | 96 | 15.4 (4.7) | 15.0 (5.2) | t(94)= 0.43 | | .84 |  |  |
|  | CpG 47.55 | 113 | 3.2 (1.6) | 3.2 (1.7) | *W=* 1592.5 | | .99 |  | 99 | 3.8 (1.7) | 3.3 (2.6) | *W=* 1538 | | .14 |  |  |
|  | CpG 60.61 | 113 | 1.1 (1.7) | 1.14 (0.94) | *W=* 1368 | | .36 |  | 99 | 1.2 (1.4) | 1.2 (2.0) | *W=* 1281 | | .84 |  |  |
|  | CpG 62 | 110 | 1.4 (1.4) | 1.4 (1.3) | *W=* 1536 | | .99 |  | 92 | 3.2 (2.9) | 2.1 (1.6) | *W=* 1350.5 | | .14 |  |  |
|  | CpG 63.64 | 113 | 1.0 (0.8) | 1.4 (1.1) | *W=* 1215.5 | | .07 |  | 99 | 1.5 (1.1) | 1.4 (1.1) | *W=* 1318.5 | | .84 |  |  |
|  | CpG 67 | 113 | 0.2 (0.6) | 0.2 (0.7) | *W=* 1565 | | .99 |  | 99 | 0.5 (1.0) | 0.3 (0.7) | *W=* 1281 | | .84 |  |  |
|  | CpG 68.69 | 113 | 2.0 (2.0) | 1.9 ( 1.2) | *W=* 1436 | | .59 |  | 98 | 2.9 (1.7) | 2.8 (1.9) | *W=* 1276 | | .84 |  |  |
|  | CpG 76.77.78 | 113 | 4.4 (3.2) | 4.7 (2.3) | *W=* 1283 | | .19 |  | 99 | 4.6 (2.2) | 4.7 (1.7) | *W=* 1196 | | .88 |  |  |
|  | CpG 79.80 | 113 | 2.4 (1.4) | 2.6 (1.0) | *W=* 1292 | | .19 |  | 99 | 3.0 (1.5) | 3.3 (1.3) | *W=* 1148 | | .84 |  |  |
| ^a^ Maternal CpG units excluded from analyses following quality control  All analyses tested two-sided. CM= childhood maltreatment. FDR= False discovery rate. | | | | | | | |  |  |  |  |  |  | |  | |
